# Hepatocyte Toll‐like receptors contribute to the hepcidin inflammatory response to pathogens and pathogen‐derived ligands

**DOI:** 10.1002/hem3.70096

**Published:** 2025-04-03

**Authors:** Katharina Bonitz, Silvia Colucci, Ruiyue Qiu, Sandro Altamura, Richard Sparla, Katja Mudder, Stefan Zimmermann, Matthias W. Hentze, Martina U. Muckenthaler, Oriana Marques

**Affiliations:** ^1^ Department of Pediatric Oncology, Hematology and Immunology Hopp Children's Cancer Center (KiTZ), University Hospital Heidelberg Heidelberg Germany; ^2^ Molecular Medicine Partnership Unit (MMPU) EMBL University of Heidelberg Heidelberg Germany; ^3^ Department of Infectious Diseases, Microbiology and Hygiene University Hospital Heidelberg Heidelberg Germany; ^4^ European Molecular Biology Laboratory (EMBL) Heidelberg Germany; ^5^ Translational Lung Research Center Heidelberg (TLRC), German Center for Lung Research (DZL) University of Heidelberg Heidelberg Germany; ^6^ German Centre for Cardiovascular Research (DZHK), Partner Site Heidelberg/Mannheim Heidelberg Germany

## Abstract

Iron restriction is a critical pathomechanism underlying the Anemia of Inflammation and an innate immune response limiting the replication of extracellular pathogens. During infections, innate immune cells detect pathogen‐associated molecular patterns (PAMPs) and produce proinflammatory cytokines. Among these, interleukin (IL)‐6 is detected by hepatocytes, where it activates the production of the iron‐regulated hormone hepcidin that inhibits iron export from macrophages. Consequently, macrophages accumulate iron and hypoferremia (low plasma iron) develops. Whether Toll‐like receptors (TLRs) expressed on hepatocytes directly recognize PAMPs and contribute to hepcidin upregulation is still an open question. Stimulation of primary murine hepatocytes with a panel of PAMPs targeting TLRs 1–9 revealed that the TLR5 ligand flagellin and the TLR2:TLR6 ligand FSL1 upregulated hepcidin. Hepcidin was also induced upon treatment with heat‐killed *Staphylococcus aureus* (HKSA) and *Brucella abortus* (HKBA). The hepcidin response to flagellin, FSL1, HKSA, and HKBA started at an early time point, was independent of autocrine regulation by IL‐6, and occurred through the TLR‐mitogen‐activated protein kinase (MAPK) axis. By analyzing a macrophage:hepatocyte co‐culture, we additionally show that the hepcidin response was dependent on TLR2:TLR6 expression in hepatocytes and independent of macrophage cytokine secretion. Ex vivo liver perfusion of mice with FSL1 and HKSA further revealed that PAMPs and pathogens can pass the sinusoidal barrier and reach hepatocytes to cause hepcidin upregulation in a TLR2:TLR6‐dependent manner. We conclude that hepatocytes can directly recognize PAMPs and pathogens and promote hepcidin upregulation in a macrophage and cytokine‐independent manner. This positions hepatocytes in the spotlight as potential direct drivers of iron restriction.

## INTRODUCTION

The term nutritional immunity was initially coined by Eugene Weinberg in 1975 and refers to vertebrate host strategies that limit metal availability for the proliferation of extracellular pathogens.^1^ Notably, iron, the most widely used trace metal in biological processes, is essential for the proliferation and pathogenicity of microorganisms. During infections, the host sequesters iron in macrophages to prevent its acquisition by pathogens.^2^ As a consequence, hypoferremia (reduced serum iron levels) develops. Despite protecting against siderophilic pathogens, this condition may lead to the Anemia of Inflammation, if prolonged over time.^3^

The underlying pathomechanism involves transcriptional activation of hepcidin (gene name: *Hamp*),^4^, ^5^ the master regulator of systemic iron flows. Hepcidin binds to its receptor ferroportin, promoting its internalization and degradation.^6^ Hepcidin‐mediated ferroportin degradation in duodenal enterocytes and reticuloendothelial macrophages inhibits iron export to the plasma and partly explains the establishment of hypoferremia.^7^ Innate immune cells recognize pathogens or pathogen‐associated molecular patterns (PAMPs) via pathogen recognition receptors (PRRs) and secrete cytokines that stimulate *Hamp* transcription in hepatocytes. Specifically, interleukin (IL)‐6 and IL‐1β are strong *Hamp* inducers in cultured hepatocytes and mice.^4^, ^5^, ^8^, ^9^, ^10^ Additionally, PAMPs, such as bacterial cell wall components, also promote *Hamp* transcription in macrophages through Toll‐like receptor (TLR) activation.^11^

Hepatocytes play an active role in systemic immune responses. They produce proteins that participate in pathogen removal (e.g., complement proteins and other opsonins such as C‐reactive protein), promote iron sequestration (e.g., hepcidin and lipocalin‐2), and induce systemic immunological responses by secreting cytokines and chemokines.^12^ Importantly, hepatocytes express PRRs, including TLRs.^12^, ^13^ Whether TLR activation by PAMPs in hepatocytes directly promotes *Hamp* upregulation and contributes to hypoferremia, however, is not fully understood.

In this study, we show that TLR2:TLR6 and TLR5 stimulation by their respective ligands or heat‐killed bacteria promotes *Hamp* upregulation in murine hepatocytes independently of autocrine and paracrine control via IL‐6 and IL‐1β. The observation that pathogens and pathogen‐derived ligands can cross hepatic sinusoids, reach hepatocytes, and thereby promote *Hamp* upregulation, within a short period, places hepatocytes in the spotlight as potential direct drivers of iron restriction during infection.

## MATERIALS AND METHODS

### Mice

C57BL/6N wild‐type (WT), *Toll‐like receptor 2* knock‐out (KO) (*Tlr2*KO, B6.129‐*Tlr2* ^*tm1Kir*^), *Toll‐like receptor 6* KO (*Tlr6*KO, B6.129‐*Tlr6* ^*tm1Aki*^), and *Toll‐like receptor 2/6* KO (*Tlr2/6*KO, generated by mating *Tlr2* with *Tlr6*KO mice) mice were housed in the specific pathogen‐free barrier at the Interfakultäre Biomedizinische Forschungseinrichtung animal facility at the University of Heidelberg (Germany). Animals were provided with a constant light‐dark cycle and maintained on a standard mouse diet containing 200 ppm iron with *ad libitum* access to food (LASQCdiet® Rod18, LASvendi, Soest, Germany) and water. Animal experiments were approved by the Regierungspräsidium Karlsruhe (T47/22 and T58/21). Mice aged 8–12 weeks were used for the preparation of primary hepatocytes. WT and *Tlr2/6*KO mice were used for the generation of bone marrow‐derived macrophages (BMDMs) and primary hepatocytes were age‐ and sex‐matched. Male WT and *Tlr2/6*KO mice aged 8–12 weeks were used for *ex vivo* perfusion experiments.

### Preparation of primary cells

#### Isolation of primary murine hepatocytes

Primary murine hepatocytes were prepared by a two‐step perfusion method, as previously described.^14^ Briefly, after euthanasia, the cava vein was cannulated with a 27G × 3/4″ needle and a perfusion speed of 5 mL/min. Livers were perfused with pre‐heated liver perfusion (Gibco), followed by liver digest medium (Life Technologies). The liver capsule was disrupted mechanically in hepatocyte wash medium (Gibco) and passed through 100 and 70 µm strainers to obtain a single‐cell suspension of liver cells. Hepatocytes were pelleted by centrifugation twice (50 g, 2 min) and resuspended in William's E medium (Gibco) supplemented with 4% fetal bovine serum (FBS) (Gibco) and 1% penicillin/streptomycin (Sigma). The supernatant, containing non‐parenchymal cells, was discarded. Hepatocytes were seeded in collagen (Gibco)‐coated plates at a cell density of 70.000 cells/cm^2^ for mono‐ and co‐culture and washed with Dulbecco's phosphate‐buffered saline solution (PBS, Sigma) to remove debris, after 4 h unless stated otherwise in figure legends. Cells were kept in culture for a maximum of 48 h. Hepatocytes were maintained at 37°C and 5% of carbon dioxide (CO_2_).

#### Isolation of BMDMs

BMDMs were generated as described before.^15^ In summary, cells were flushed from the femurs of C57BL/6N WT and *Tlr2/6*KO mice with PBS, filtered through a 70 µM strainer and plated at a concentration of 73.000 cells/cm^2^ in RPMI 1640+ L‐Glutamine (Gibco) supplemented with 10% FBS (Gibco), 10 ng/mL macrophage colony‐stimulating factor 1 (PeproTech) and 1% penicillin/streptavidin (Sigma). After 4 days, non‐adherent cells were removed by washing with PBS and the medium was replaced daily until cells were used. BMDMs were maintained at 37°C and 5% CO_2_. BMDMs were detached on day 6 using Accutase (Sigma) and plated with hepatocytes at different ratios (M0:HC 1:3, 1:6, and 1:12) or replated in monoculture. Cells were allowed to attach for 24 h before stimulation.

### *Ex vivo* perfusion

Mouse livers were perfused with 35 mL of pre‐heated liver perfusion medium (Gibco) supplemented with saline solution (vehicle), 1 µg FSL1 (Invivogen) or 10^8^ particles of heat‐inactivated *Staphylococcus aureus* (Invivogen). Livers were either prepared for whole tissue RNA isolation and fixation for histology or hepatocyte isolation, as described earlier. Isolated hepatocytes resuspended in William's E medium were plated for analysis of gene expression at designated time points.

### Cell culture treatments

#### TLR ligands and cytokines

Cells were treated with the following TLR ligands (all at 100 ng/mL): Pam3CSK4 (TLR2:1, Invivogen), PGN‐SA (TLR2, Invivogen), Poly I:C (TLR3, Invivogen), LPS (TLR4, Sigma‐Aldrich), FLA‐ST (TLR5, Invivogen), FSL1 (TLR2:TLR6, Invivogen), R848 (TLR7:8, Invivogen), and ODN (TLR9, Invivogen). Heat‐inactivated *S. aureus* (Invivogen) and *Brucella abortus* were used at a multiplicity of infection 1:100. The batch of *B. abortus* strain 544 was prepared at the Department of Medical Microbiology and Hygiene, Universitätsklinikum Heidelberg. The antigen preparation was inactivated using a BD ProbeTec Lysolyzer oven (Becton Dickinson) by heating the tubes to 105°C for 30 minutes. Successful inactivation was confirmed by the absence of growth on solid (Columbia blood agar, BD) and liquid media (brain heart broth, BD) for 14 days (35°C). TNF, IL‐6, and IL‐1β (Peprotech) were used at 20 ng/mL and BMP2 (Peprotech) and Insulin (Sigma‐Aldrich) at 100 ng/mL. Cell stimulation was performed for 4 h before collection unless stated otherwise in figure legends.

#### RNA interference

RNAi‐mediated knockdown of *Il6ra* (s68295, Thermo Scientific), *l1r1* (s68244, Thermo Scientific), and *Smad4* (s132210, Thermo Scientific) was performed with Lipofectamine RNAiMAX according to the manufacturer's protocol. The Silencer Select Negative Control #2 siRNA (4390847, Thermo Scientific) was used to provide a baseline to compare siRNA‐treated samples.

#### Pharmacological inhibitors

Cells were pre‐treated for 1 h with the inhibitors listed in Table 1, followed by incubation with the ligands described earlier.

**Table 1:** List of pharmacological inhibitors used and respective concentrations.

Inhibitor	Concentration (µM)	Company
Actinomycin D	1	A9415, Sigma‐Aldrich
BI605906	10	Biomol
JNK Inhibitor VIII	20	Hycultec
SB203580	20	InvivoGen
SCH772984	10	Biomol
SP600125	50	InvivoGen
U0126	10	Cell Signaling
Wortmannin	1	Sigma‐Aldrich
Ulixertinib	5	Biomol

### RNA extraction, reverse transcription PCR, and quantitative real‐time PCR (qPCR)

Total RNA was isolated from primary hepatocytes using the RNA Mini kit (Bio&SELL) according to the manufacturer's instructions. Retrotranscription was performed with 1 µg of total RNA in a 25 µL reaction using RevertAid H Minus Reverse Transcriptase (Thermo Scientific) and random oligoprimers (Invitrogen). SYBR‐green qPCR was performed using the ABI StepONE Plus real‐time PCR system (Applied Biosystems) with the primers listed in Table 2. The mRNA expression of the gene of interest was normalized to the housekeeping gene *Rpl19*, and data were analyzed using the ΔΔCt method.^16^

**Table 2:** List of primers used for qPCR analysis.

Gene name	Forward primer	Reverse primer
*Adgre1* (F4/80)	GGAGGACTTCTCCAAGCCTATT	AGGCCTCTCAGACTTCTGCTT
*G6pc1*	TCTGTCCCGGATCTACCTTG	GAAAGTTTCAGCCACAGCAA
*Hamp*	ATACCAATGCAGAAGAGAAGG	AACAGATACCACACTGGGAA
*Il1b*	GCAACTGTTCCTGAACTCAACT	ATCTTTTGGGGTCCGTCAACT
*Il1r1*	CCGTGAACAACACAAATGGAGA	TCAATCTCCAGCGACAGCAG
*Il6*	GCTACCAAACTGGATATAATCAGGA	CCAGGTAGCTATGGTACTCCAGAA
*Il6ra*	TCACTGTGCGTTGCAAACAG	TACCACAAGGTTGGCAGGTG
*Tnf*	TGCCTATGTCTCAGCCTCTTC	GAGGCCATTTGGGAACTTCT

### Western blot

Protein lysates were obtained by homogenizing samples in RIPA buffer supplemented with protease and phosphatase inhibitors (Roche). Protein concentration was determined using the Pierce BCA Protein Assay (Thermo Scientific). Thirty to fifty µg of protein were separated using 10% sodium dodecyl‐sulfate polyacrylamide gel electrophoresis (SDS‐PAGE) followed by analysis with the antibodies listed in Table 3. Western blot images were acquired and quantified with the Vilber Lourmat Fusion‐FX Chemiluminescence System.

**Table 3:** List of antibodies used for western blot.

Antibody	Dilution	Reference, Company
Vinculin	1:1000	V4505, Sigma‐Aldrich
Phospho‐p38 MAPK	1:1000	4511, Cell Signaling
Phospho‐SAPK/JNK	1:1000	9251, Cell Signaling
SAPK/JNK	1:1000	9252, Cell Signaling
Phospho‐44/42 MAPK (ERK1/2)	1:1000	4370, Cell Signaling
44/42 MAPK (ERK1/2)	1:1000	4695, Cell Signaling
Phospho‐AKT	1:1000	4060, Cell Signaling
AKT	1:1000	9272, Cell Signaling
pSMAD5	1:1000	ab92698, Abcam
pSTAT3	1:1000	9138, Cell Signaling

### Cytokine/chemokine measurements

Supernatants from cell cultures were collected and frozen at −80°C until use. Murine chemokine (C‐X‐C motif) ligand 1 (CXCL1), Free Active Transforming Growth Factor β 1 (TGF‐β1), IL‐18, IL‐23, C‐C motif chemokine 22 (CCL22), IL‐10, IL‐12p70, IL‐6, Tumor Necrosis Factor α (TNF‐α), Granulocyte Colony‐Stimulating Factor (G‐CSF), Thymus and Activation‐Regulated Chemokine (TARC), IL‐12p40, and IL‐1β were measured using the LEGENDplex Mouse Macrophage/Microglia Panel (BioLegend) according to the manufacturer's instructions. Data analysis was performed with the LEGENDplex Data Analysis Software Suite.

### Immunohistochemistry and Gram staining

Liver sections were fixed in 10% neutral buffered formalin (Sigma) overnight at 4°C, dehydrated and embedded in paraffin. Three μm tissue microtome sections were subjected to immunohistochemistry for pERK (4370, Cell Signalling) and pJNK (sc‐6254, Santa Cruz Biotechnology) with the Vectastain ABC kit (Vector Labs), after blocking of endogenous peroxidases with 3% H_2_O_2_ (Sigma Aldrich) and antigen retrieval with the Citra Plus solution (BioGenex). Immunoreactivity was developed using the Vector AEC substrate (Vector Labs), and then slides were counterstained with Hematoxylin (Sigma‐Aldrich). Heat‐killed bacteria were detected on the livers through a modified Brown & Brenn Gram staining (Biorbyt), according to the manufacturer's instructions. Briefly, liver sections were deparaffinized and hydrated, followed by incubation with the gentian violet solution for 2 min. Sections were then washed with a couple drops of lugol's iodine solution and left to incubate with the latter solution for 1 min. Excess solution was removed by rinsing with water. Sections were then decolorized and rinsed with water. Slides were mounted with the VectaMount AQ mounting medium (Vector Labs). Images were digitally acquired with a Nikon Ni‐E microscope, using the Nikon NIS‐Elements software.

### RNA‐seq data set

RNA‐seq data for murine hepatocyte samples were obtained from the Gene Expression Omnibus database (GSM2540245 and GSM2540246),^17^ representing two biological replicates. Expression levels (fragments per kilobase of transcript per million mapped reads, FPKM) of TLR genes (*Tlr1–Tlr9*) were extracted and normalized by dividing each gene's FPKM value by the total FPKM of all TLR genes within the same sample using R (version 4.3.3). Data visualization was performed using Prism v8 (GraphPad Software).

### Statistical analyses

Data are shown as mean ± standard deviation (SD) and representative of, at least, three independent experiments (biological repeats). Statistical analyses were performed using Prism v8 (GraphPad Software). The two‐tailed, Student's *t*‐test, One‐way or two‐way ANOVA in combination with Dunnett's multiple comparison test or Sidak's multiple comparison test were used, whenever appropriate, and indicated in figure legends. Significance is indicated as *^/#^ *p* < 0.1; **^/##^ *p* < 0.01; ***^/###^ *p* < 0.001; and ****^/####^ *p* < 0.0001.

## RESULTS

### TLR2:TLR6 activation induces *Hamp* in primary hepatocytes

**Figure 1:**
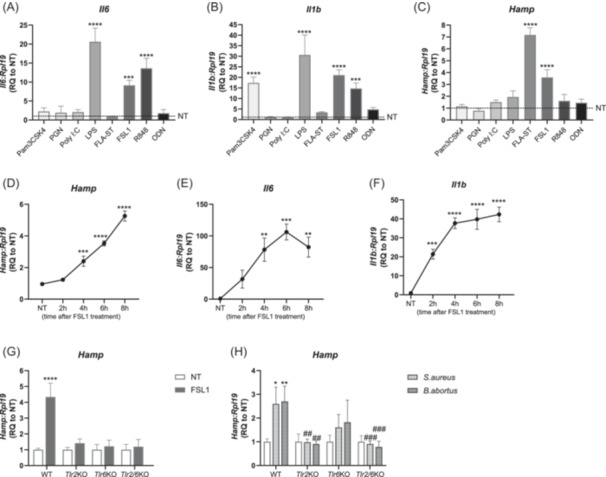
**TLR2 and TLR5 activation induces** ***Hamp*** **mRNA expression in primary hepatocytes. (A–C)** Primary murine hepatocytes were stimulated for 4 h with 100 ng/mL of TLR1‐9 specific ligands. The mRNA expression of **(A)** *Il6*, **(B)** *Il1b*, and **(C)** *Hamp* were analysed by qRT‐PCR analysis. **(D–F)** Primary murine hepatocytes were stimulated for the indicated time points with FSL1. The mRNA expression of **(D)** *Hamp*, **(E)** *Il6*, and **(F)** *Il1b* were analysed by qRT‐PCR. **(G,H)** Primary murine hepatocytes isolated from WT, *Tlr2*, *Tlr6*, and *Tlr2/6*KO mice were stimulated for 4 h with **(G)** FSL1 or **(H)** heat‐killed *S. aureus* or *B. abortus*. The mRNA expression of *Hamp* was analysed by qRT‐PCR analysis. mRNA expression data were normalized to the housekeeping gene *Rpl19*. One representative experiment with four technical replicates. Data are reported as mean ± SD. One‐way ANOVA followed by Dunnett's multiple comparison test/two‐way ANOVA, followed by Sidak's multiple comparisons test. *^/#^Refers to comparisons with the respective non‐treated controls and with the matched treatment in WT controls, respectively: *^/#^ *p* < 0.05, **^/##^ *p* < 0.01, ***^/###^ *p* < 0.001, ****^/####^ *p* < 0.0001. NT, non‐treated; RQ, relative quantification; Tlr, Toll‐like receptor; WT, wild‐type.

To explore if hepatocytes can directly detect pathogens and/or their associated ligands and regulate *Hamp* levels in a cell‐autonomous manner, we incubated primary hepatocytes with pathogen‐derived ligands that specifically activate homo‐ and hetero‐ dimers of TLR 1‐9 (Table S1). We show that stimulation with LPS (TLR4), FSL1 (TLR2:TLR6), or R848 (TLR7:TLR8) increased the mRNA expression of *Il6* and *Il1b*, while stimulation with Pam3CSK4 (TLR2:TLR1) only increased *Il1b* mRNA expression (Figure 1A,B), suggesting that these TLRs expressed in hepatocytes can elicit downstream pro‐inflammatory responses. Among these TLRs, only TLR2:TLR6 hetero‐dimer activation by FSL1 increased *Hamp* mRNA levels (Figure 1C). *Hamp* mRNA levels were significantly higher 4 h after stimulation with FSL1, and concurrent with the upregulation of pro‐inflammatory cytokines, such as *Il6* and *Il1b* (Figure 1D–F). In contrast, TLR5 activation by FLA‐ST (flagellin isolated from *Salmonella typhimurium*) strongly induced *Hamp* mRNA expression, despite the absence of a canonical pro‐inflammatory response (Figure 1A–C). We found that hepatocytes express TLRs at different levels (Figure S1A), with *Tlr5* and *Tlr2* showing the highest expression and correlating with increased *Hamp* transcription (Figure 1C). TLRs expressed at lower mRNA levels (e.g., *Tlr4* and *Tlr7/8*), despite not affecting *Hamp* expression following receptor stimulation, still elicited robust *Il6* and *Il1b* responses (Figure 1A–C).

To confirm the specificity of FSL1 in controlling *Hamp* activation via the TLR2:TLR6 hetero‐dimer receptor, we isolated primary hepatocytes from WT, *Tlr2*KO, *Tlr6*KO, and *Tlr2/6*KO mice and exposed them to FSL1. The FSL1‐mediated *Hamp* upregulation observed in WT mice was completely abolished in hepatocytes lacking either TLR2 or TLR6 alone or TLR2 and TLR6 together (Figure 1G). The fact that hepatocytes isolated from *Tlr2/6*KO mice respond to stimulation with FLA‐ST with an upregulation of *Hamp* and *Il1b* similar to WT hepatocytes (Figure S1B,C) suggests that *Tlr2/6*KO hepatocytes do not have an intrinsic defect that hampers their responses to other TLR ligands. Importantly, *Hamp* induction was also observed in response to heat‐inactivated *S. aureus* and *B. abortus* in primary hepatocytes from WT mice, but not from *Tlr2*KO and *Tlr2/6*KO hepatocytes (Figure 1H). These data suggest a crucial involvement of TLR2 and TLR6 in the hepatocyte‐specific control of *Hamp* mRNA expression in response to respective PAMPs and Gram‐positive and Gram‐negative bacteria.

### Autocrine and paracrine signals of pro‐inflammatory cytokines do not contribute to FSL1‐mediated *Hamp* upregulation in hepatocytes

**Figure 2:**
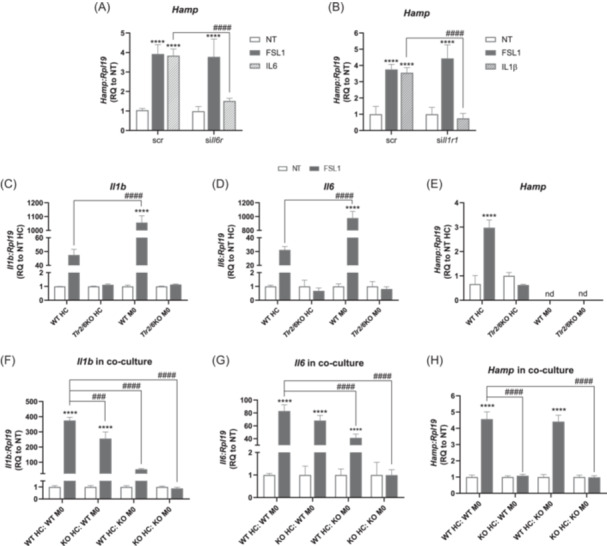
**FSL1‐mediated** ***Hamp*** **upregulation in hepatocytes is independent of autocrine or paracrine regulation by IL‐6 and IL‐1β.** RNAi for **(A)** *Il6r* and **(B)** *Il1r1* was performed in primary hepatocytes for 40 h, followed by stimulation with FSL1, IL‐6, or IL‐1β for 4 h. The mRNA expression of *Hamp* was analysed by qRT‐PCR analysis. **(C–E)** Primary cultures of hepatocytes and bone marrow‐derived macrophages derived from WT or *Tlr2/6*KO mice were stimulated with FSL1 for 4 h. The mRNA expression of (**C**) *Il1b*, (**D**) *Il6*, and (**E**) *Hamp* were analysed by qRT‐PCR analysis. **(F–H)** Co‐cultures of BMDMs and hepatocytes (ratio 1:6) derived from WT, and *Tlr2/6*KO mice were stimulated with FSL1 for 4 h. The mRNA expression of (**F**) *Il1b*, (**G**) *Il6*, and (**H**) *Hamp* was analysed by qRT‐PCR analysis. mRNA expression data were normalized to the housekeeping gene *Rpl19*. One representative experiment with four technical replicates. Data are reported as mean ± SD. Two‐way ANOVA, followed by Sidak's and Dunnett's multiple comparison tests. *^/#^Refers to comparisons with the respective non‐treated controls and with the matched treatment in scr/WT controls, respectively: ****^/####^ *p* < 0.0001. NT, non‐treated; RQ, relative quantification; scr, scrambled; Tlr, Toll‐like receptor; WT, wild‐type.

To address whether IL‐6 and IL‐1β that are induced in FSL1‐treated primary hepatocytes are required for the TLR2:TLR6 mediated *Hamp* response (Figure 1D–F), we knocked down the IL‐6 and IL‐1β receptors (IL6RA and IL1R1) by RNAi in primary hepatocytes and exposed them to FSL1 or the corresponding activating ligands, as a control. We observed a silencing efficiency superior to 80% (Figure S2A,B), which was sufficient to abrogate *Hamp* upregulation in response to IL‐6 or IL‐1β treatment (Figure 2A,B). Despite that, the ability of FSL1 to induce *Hamp* expression was maintained. To test if there was a compensatory factor for one receptor in the absence of the other, we silenced, simultaneously, *Il6r* and *Il1r1* and then stimulated the hepatocytes with IL‐6, IL‐1β, or FSL1. Despite a reduced silencing efficiency with the double knockdown compared to the single knockdown of the two receptors (Figure S2C,D), FSL1‐mediated *Hamp* upregulation was not affected by concurrent knockdown of *Il6r* and *Il1r1* (Figure S2E), suggesting that even when the pathways downstream of IL‐6R and IL‐1R are simultaneously affected, hepcidin upregulation in response to FSL1 is not. FSL1 also did not activate bone morphogenetic protein/small mothers against decapentaplegic (BMP/SMAD) or Janus kinase/signal transducers and activators of transcription (JAK/STAT) signaling, as indicated by a lack of SMAD5 and STAT3 phosphorylation, respectively, during the 4‐h stimulation period (Figure S2F). In contrast, IL‐6 and IL‐1β‐mediated *Hamp* induction in hepatocytes requires active BMP/SMAD signaling.^18^, ^19^, ^20^ Additionally, *Smad4* silencing did not impair the capacity of hepatocytes to upregulate *Hamp* in response to FSL1 (Figure S2G,H), supporting the conclusion that *Hamp* activation by FSL1 is independent of BMP/SMAD signaling. The system was tested further by treating primary hepatocytes with IL‐6, IL‐1β, or BMP2, alone or together with FSL1. The addition of FSL1 to hepatocytes simultaneously treated with IL‐6 significantly increased *Hamp* mRNA expression (Figure S2I). Similar results were observed for co‐treatment of FSL1 and BMP2. This suggests that FSL1 additively affects the *Hamp* response with IL‐6 and BMP2, and that these stimuli target *Hamp* through different pathways. In contrast, hepatocytes treated with IL‐1β and FSL1 displayed similar (despite tendentially higher) *Hamp* levels than those treated with IL‐1β alone and significantly higher *Hamp* levels than those treated with FSL1 alone. This suggests that while FSL1 and IL‐1β may partially share regulatory pathways for *Hamp* upregulation, IL‐1β likely also activates additional, independent mechanisms, as evidenced by the higher *Hamp* levels in the combined treatment compared to FSL1 alone.

We next investigated the responses of 13 additional cytokines in the cell culture medium of primary hepatocytes treated with FSL1. Remarkably, only the CXC Motif Chemokine Ligand 1 (CXCL1) was substantially increased in the medium upon FSL1 treatment, while most of the other cytokines (including IL‐6 and IL‐1 β) remained below the threshold of detection (Table S2). Importantly, primary hepatocytes treated with increasing concentrations of CXCL1, for different time intervals, did not display significantly altered *Hamp* levels (Figure S3A). Therefore, it is unlikely that the pro‐inflammatory cytokines/chemokines detected in the supernatant promote *Hamp* upregulation in hepatocytes treated with FSL1 in an autocrine manner.

In the liver, macrophages neighboring hepatocytes produce cytokines following exposure to pathogens and/or PAMPs. Therefore, we established a co‐culture system using BMDMs (M0) and primary hepatocytes, in ratios of 1:3, 1:6, and 1:12 (M0:hepatocyte). Figure S3B shows that with an increasing number of macrophages, there is a corresponding rise in the expression of the macrophage marker *Adgre1* (F4/80). As expected, in co‐culture, macrophages are the main contributors to the production of *Il6* and *Il1b* (Figure S3C,D), while hepatocytes constitute the main source of *Hamp* (Figure S3E). Importantly, treatment with FSL1 does not affect the functional integrity of the co‐culture system as *Il6*, *Il1b*, and *Hamp* are upregulated and *Adgre1* expression levels indicate that the ratio between hepatocytes and macrophages remains unaffected. We next incubated mono‐ and co‐cultures of BMDMs and primary hepatocytes isolated from either WT or *Tlr2/6*KO mice with FSL1. The co‐cultures included the following combinations of cells: WT HC with WT M0, KO HC with WT M0, WT HC with KO M0, and KO HC with KO M0. We maintained a 1:6 (M0:hepatocyte) cell ratio, which reflects the relative numbers of these cell types within the murine liver.^21^ We show that FSL1 treatment of monocultures of both TLR2/6‐deficient hepatocytes and macrophages failed to upregulate *Il6*, *Il1b*, and *Hamp* mRNA levels (Figure 2C–E). In the co‐culture, cytokine mRNA production was increased in response to FSL1 when at least one cell type expressed TLR2 and TLR6 (Figure 2F,G). Importantly, *Hamp* mRNA expression was induced to similar levels when WT hepatocytes were cultivated with *Tlr2/6*KO or WT macrophages (Figure 2H). These findings highlight that the upregulation of *Hamp* 4 h following stimulation by the pathogen‐derived ligand FSL1 is not driven by autocrine or paracrine cytokine signals. Moreover, they reveal a potentially novel, cytokine‐independent pathway involving TLR2:TLR6 signaling in the regulation of *Hamp* expression in hepatocytes.

### Hepcidin expression upon pathogen and pathogen‐derived ligand treatment in hepatocytes involves the MAPK‐ERK and JNK signaling pathways

**Figure 3:**
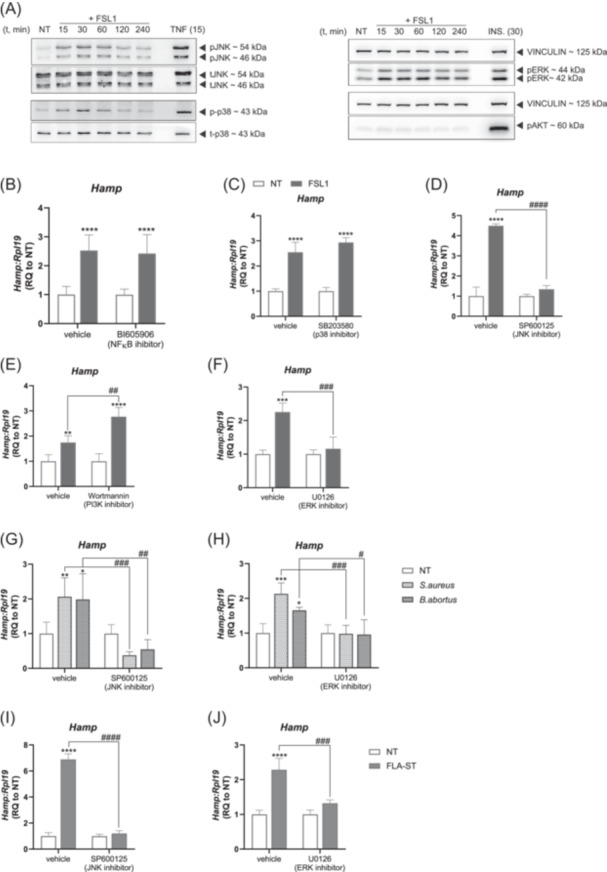
**The MAPK pathways JNK and ERK are involved in hepatocyte** ***Hamp*** **upregulation in response to PAMPs and pathogens. (A)** Primary murine hepatocytes were treated with FSL1, TNF, or Insulin for the indicated time points. Western blot analyses of phospho‐JNK, phospho‐p38, phospho‐ERK, and phosho‐AKT. Vinculin, total JNK, and total p38 were used as loading controls. Primary murine hepatocytes were pre‐treated for 1 h with **(B)** BI605906, **(C)** SB203580, **(D)** SP600125, **(E)** Wortmannin, or **(F)** U0126 and then stimulated with FSL1 for 4 h. The mRNA expression of *Hamp* was analysed by qRT‐PCR analysis. Primary murine hepatocytes were pre‐treated for 1 h with **(G, I)** SP600125 or **(H, J)** U0126 and then stimulated with **(G, H)** heat‐killed *S. aureus*, *B. abortus*, or **(I, J)** FLA‐ST for 4 h. The mRNA expression of *Hamp* was analysed by qRT‐PCR analysis. mRNA expression data were normalized to the housekeeping gene *Rpl19*. One representative experiment with four technical replicates. Data are reported as mean ± SD. Two‐way ANOVA, followed by Sidak's and Dunnett's multiple comparison tests. */^#^Refers to comparisons with the respective non‐treated controls and with the matched treatment in vehicle controls, respectively: *^/#^ *p* < 0.05, **^/##^ *p* < 0.01, ***^/###^ *p* < 0.001, ****^/####^ *p* < 0.0001. INS, Insulin; NT, non‐treated; p, phospho; RQ, relative quantification; t, total.

We next examined the direct impact of TLR2:TLR6 activation on *Hamp* transcription in hepatocytes. To confirm that FSL1‐mediated *Hamp* upregulation occurs mainly at the transcriptional level, we treated hepatocytes with the inhibitor of *de novo* transcription Actinomycin D, followed by stimulation with FSL1. Actinomycin D blocked *Hamp* upregulation, suggesting that *de novo* transcription is required for the *Hamp* response (Figure S4A). We further show an increased phosphorylation of p38, JNK, and ERK, as well as an increased expression of the NFκB target gene *Tnf* (Figures 3A and S4B) within 15 min of FSL1 treatment. To assess which of these pathways is functionally relevant for the hepcidin response, we systematically incubated primary hepatocytes with FSL1 in the presence of inhibitors targeting the NFκB (BI605906), p38 (SB203580), JNK (SP600125 and JNKiVIII), PI3K‐AKT (Wortmannin), and MAPK‐ERK (U0126, Ulixertinib, and SCH772984) signaling branches (Figures 3B–F and S4B–I). Interestingly, only the inhibition of the JNK and MAPK‐ERK signaling pathways prevented *Hamp* upregulation in response to FSL1. In contrast, FSL1‐mediated upregulation of pro‐inflammatory cytokines was affected by inhibition of the JNK, ERK, and NFκB pathways (Figure S4B,J–L). The concurrent activation of the JNK and ERK pathways appears to be necessary for *Hamp* upregulation to occur given that, from the set of TLR2 ligands tested, only FSL1 is capable of promoting the simultaneous phosphorylation of JNK and ERK together with *Hamp* upregulation (Figures 1C and S4M). The JNK and ERK pathways are equally important for the *Hamp* response driven by heat‐killed *S. aureus* or *B. abortus*, as highlighted by the absence of *Hamp* upregulation upon treatment with specific pathway inhibitors (Figure 3G,H).

Figure 1C shows that the pathogen‐derived ligand flagellin (FLA‐ST) triggers *Hamp* transcription via TLR5, independently of IL‐6 and IL‐1β production (Figure 1A,B). As observed for FSL1, FLA‐ST‐mediated *Hamp* mRNA induction was also blocked by inhibiting *de novo* transcription with Actinomycin D (Figure S4N). Therefore, we treated primary hepatocytes with FLA‐ST in the presence or absence of SP600125 or U0126 to inactivate JNK and MAPK‐ERK signaling, respectively. Remarkably, *Hamp* induction by TLR5 activation was completely abolished (Figure 3I,J). Altogether, these findings reveal the pivotal role of the JNK and MAPK‐ERK signaling pathways in the regulation of *Hamp* in response to heat‐inactivated pathogens and derived ligands recognized by TLR2:TLR6 and TLR5.

### PAMPs and pathogens can cross the liver sinusoidal endothelial cell barrier and directly upregulate *Hamp* in hepatocytes

**Figure 4:**
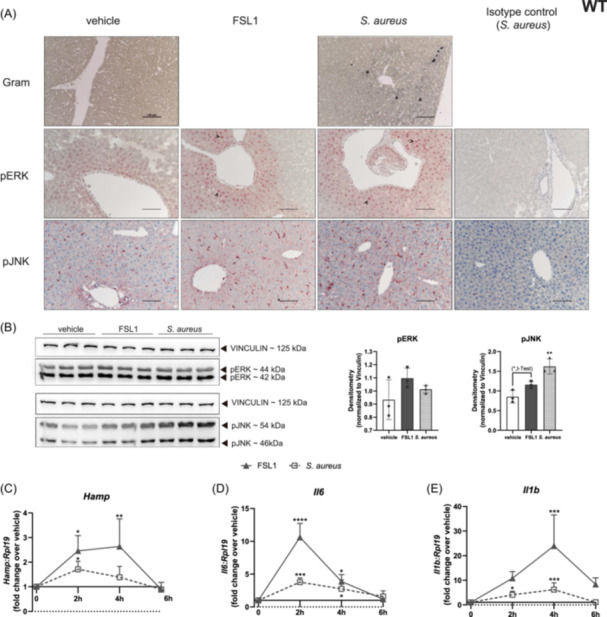
***Ex vivo* liver perfusion with FSL1 or heat‐killed** ***S. aureus*** **induces hepatocyte** ***Hamp*** **mRNA expression. (A)** Livers from WT mice were perfused with FSL1 or heat‐killed *S. aureus* and then further processed. Liver slices were fixed in 4% PFA, embedded in paraffin, and cut at 5 µm thickness. Sections were stained for Gram or processed for immunohistochemical analysis of phospho‐ERK and phospho‐JNK. Asterisks indicate bacterial aggregates and arrows nuclear immunostaining. **(B)** After perfusion, liver pieces were collected. Western blot analyses of phospho‐JNK and phospho‐ERK. Vinculin was used as a loading control and for data normalization. The *t*‐test and one‐way ANOVA followed by Dunnett's multiple comparison test. *Refers to comparisons with the non‐treated control. **(C–E)** Hepatocytes isolated from the livers perfused with FSL1 or heat‐killed *S. aureus* were plated and analyzed at the indicated time points. The mRNA expression of **(C)** *Hamp*, **(D)** *Il6*, and **(E)** *Il1b* were analysed by qRT‐PCR analysis. mRNA expression data were normalized to the housekeeping gene *Rpl19*. One representative experiment with four technical replicates. Data are reported as mean ± SD. Two‐way ANOVA, followed by Sidak's multiple comparison test. *Refers to comparisons with the matched treatment and time point in vehicle controls: *p < 0.05, **p < 0.01, ***p < 0.001, ****p < 0.0001. p, phospho; RQ, relative quantification.

Given the fact that PAMPs or pathogens in circulation have to pass the liver sinusoids to exert their effect directly on hepatocytes, we performed *ex vivo* liver perfusion with FSL1 or heat‐killed *S. aureus* and collected the tissue for further analysis. Gram staining via gentian violet showed the accumulation of heat‐killed *S. aureus* bacterial aggregates (Figure 4A) beyond the sinusoids and in close contact with hepatocytes. Consistent with our results in cultured primary hepatocytes (Figure 3A) liver perfusion with FSL1 or *S. aureus* increased phosphorylation of ERK, as detected by immunohistochemical staining of centrolobular hepatocytes (Figure 4A). In contrast, increased JNK phosphorylation was only detectable in the total liver of mice perfused with *S. aureus* but not in hepatocytes by immunohistochemistry (Figure 4A,B). Importantly, cultured hepatocytes from FSL1 and *S. aureus* perfused livers displayed significantly higher *Hamp* mRNA levels than vehicle‐perfused livers, starting from 2 h after perfusion (Figure 4C). The response normalized after 6 h in culture. The mRNA expression of *Il6* and *Il1b* followed similar dynamics, with a clear peak of increased expression in hepatocytes from PAMP‐perfused livers at 2 and 4 h after culturing, respectively (Figure 4D,E). These results suggest that PAMPs can pass the sinusoidal barrier and reach hepatocytes causing *Hamp* upregulation.

The hepcidin response to FSL1 and *S. aureus* in hepatocytes isolated from perfused livers depends on TLR2 and TLR6, as *Hamp* mRNA levels failed to be upregulated in hepatocyte cultures isolated from *Tlr2/6*KO mice (Figure S5C). The observed lack of *Hamp* upregulation is unlikely due to a different permeability of liver sinusoids in *Tlr2/6*KO mice, as Gram‐positive bacterial aggregates were also detected in the *S. aureus* perfused livers (Figure S5A). In addition, significantly increased JNK, but not ERK, phosphorylation was observed in the livers of *Tlr2/6*KO mice perfused with *S. aureus* (Figure S5A,B). Despite the lack of a hepcidin response, hepatocytes from the livers of *Tlr2/6*KO mice perfused with *S. aureus* still presented increased *Il6* and *Il1b* mRNA levels. This suggests that other hepatocyte receptors may mediate a response to *S. aureus* to increase cytokine mRNA levels. In contrast, TLR2:TLR6 are essential for *Hamp* upregulation. Taken together, these results reveal a crucial role for TLR2:TLR6 in hepatocytes in controlling *Hamp* levels in response to PAMPs and pathogens that reach the liver via circulation.

## DISCUSSION

It is widely accepted that infection‐triggered hypoferremia is caused by the following steps: (1) innate immune cells recognize PAMPs and pathogens and secrete cytokines that promote the production of acute‐phase proteins, such as hepcidin, in the liver.^2^ (2) Hepcidin transcription is increased by macrophage‐derived IL‐6 that binds to the IL‐6R and activates the JAK/STAT signaling pathway in hepatocytes.^8^, ^9^ (3) Hepcidin is secreted from hepatocytes and binds the iron exporter ferroportin expressed in duodenal enterocytes and macrophages to trigger its degradation.^4^, ^6^ In addition, ferroportin transcription is repressed in macrophages in a TLR2/6 and NFκB‐dependent manner.^22^, ^23^ (4) As a consequence, iron release from iron‐recycling macrophages is blocked, causing hypoferremia in the plasma. (5) Iron accumulation in macrophages may further enhance cytokine secretion.^24^ Iron restriction is part of the host‐defense mechanisms against extracellular pathogens that rely on iron for proliferation. Here we show that hepatocytes can stimulate hepcidin transcription in response to PAMPs and pathogens via TLR2:TLR6 and TLR5 in a cell‐intrinsic manner, independently of autocrine and paracrine control by key inflammatory cytokines. This finding advances our knowledge about the role of hepatocytes as critical immunological players.

By screening primary murine hepatocytes for their responsiveness to TLR 1‐9 ligands at constant concentrations and a short time point (4 h), we were able to observe increased *Hamp* mRNA levels upon stimulation with the TLR5 ligand flagellin (FLA‐ST) and the TLR2:TLR6 ligand FSL1 (Figure 1C). In addition, heat‐killed *S. aureus* and *B. abortus* promoted *Hamp* mRNA expression in a TLR2 and partially TLR6‐dependent manner in hepatocytes at the same time point (Figure 1H), strongly supporting the involvement of TLRs in controlling *Hamp* expression in hepatocytes in response to PAMPs and pathogens. The responsiveness of hepatocytes to TLR ligands and their ability to upregulate hepcidin have been assessed in previous studies. In the study of Rodriguez et al., *Hamp* induction by PAMPs was shown to depend on IL‐6, while Lee and collaborators demonstrated *Hamp* upregulation upon stimulation with LPS via the TLR4‐MYD88‐JNK pathway.^25^, ^26^ Given that higher concentrations of PAMPs, for longer periods of time, were used in those studies, it is tempting to hypothesize that upregulation of hepcidin in hepatocytes in response to PAMPs involves sequential activation of inflammation‐related pathways. This hypothesis is supported by the fact that hepatocyte treatment with FSL1 did not activate the signaling pathways previously shown to control *Hamp* levels (i.e., BMP‐SMAD and JAK/STAT) during the stimulation period (Figure S2F).^18^, ^19^, ^20^ At early time points, TLR activation leads to concurrent hepcidin and IL‐6/IL‐1β upregulation (Figure 1D–F), followed by cytokine secretion. Once cytokine concentrations reach a certain threshold in the extracellular environment, this would then cause a secondary hepcidin upregulation. Differences between our data and previous findings regarding hepcidin induction in response to TLR4 activation^26^ and the IL‐6 dependence of the hepcidin response to PAMPs^25^ may be further explained by different sources of hepatocytes (hepatocyte cell line and primary human hepatocytes).

TLR‐driven gene expression changes depend on the cell type investigated and the specificity and amount of ligand used.^27^, ^28^ Our time‐course experiments in primary hepatocytes treated with FSL1 showed that both *Hamp* as well as pro‐inflammatory cytokine mRNA levels (*Il6* and *Il1b*) were increased at the 4‐h time point (Figure 1D–F). Interestingly, other TLR ligands, such as LPS, also promoted a canonical pro‐inflammatory response in hepatocytes but failed to upregulate *Hamp* under these conditions. Conversely, the TLR5 ligand FLA‐ST increased *Hamp* mRNA expression in hepatocytes without upregulating *Il6* and *Il1b* (Figure 1A–C). These findings suggest that TLR‐mediated *Hamp* upregulation does not strictly depend on autocrine regulation by these cytokines. This view was confirmed by the RNAi‐mediated knockdown of the receptors for IL‐6 and IL‐1β which blocked the hepcidin response to IL‐6 and IL‐1β, without affecting FSL1‐mediated *Hamp* upregulation (Figure 2A,B). Therefore, *Hamp* upregulation in hepatocytes downstream of TLR2:TLR6 activation is independent of IL‐6 and IL‐1β. This contrasts previous pioneering studies in the field that reported *Hamp* upregulation in response to PAMPs *in vitro* and *in vivo* to be mainly driven by IL‐6.^4^, ^25^ In these studies, higher concentrations of PAMPs were applied for longer periods suggesting that *Hamp* upregulation may have occurred secondarily to TLR‐mediated cytokine secretion.^5^ To minimize the effects of other factors (e.g., secreted cytokines) we chose the shortest time point in our studies at which significant *Hamp* upregulation was observed in response to TLR2:TLR6 activation with FSL1 (Figure 1D).

The relevance of the TLR2:TLR6 heterodimer in controlling the inflammatory hepcidin response to FSL1 at an early time point was confirmed in co‐culture studies with *Tlr2/6*KO hepatocytes and WT macrophages (Figure 2H). Importantly, the lack of PAMP recognition by *Tlr2/6*KO macrophages did not affect the extent of *Hamp* upregulation in the co‐culture with WT hepatocytes, despite overall decreased cytokine mRNA levels (Figure 2F,G). This result further reinforces the role of hepatocytes in initiating the *Hamp* response to PAMPs downstream of TLR activation.

While some TLRs are expressed on multiple liver cell types (e.g., hepatocytes and macrophages), TLR5 is mainly restricted to hepatocytes. TLR5 stimulation induces the most pronounced *Hamp* upregulation in our system (Figure 1C). The relevance of hepatocyte TLR5 in mediating innate immune responses is reinforced by work from Etienne‐Mesmin et al. demonstrating that mice lacking TLR5 in hepatocytes display reduced cytokine production in response to flagellin as well as reduced clearance of flagellated *Escherichia coli*.^29^ Unfortunately, hepcidin and serum iron levels were not analyzed in this study. The non‐overlapping expression of TLR5 in liver cell types ^28^, ^30^ further supports the importance of hepatocyte TLR5‐mediated PAMP recognition and downstream inflammatory responses in the liver.

JNK and ERK activation downstream of TLR activation in hepatocytes is required for the *Hamp* upregulation in response to FSL1, FLA‐ST, and the heat‐killed bacteria *S. aureus* and *B. abortus* (Figure 3). Of note, the JNK pathway has been previously identified as a regulator of hepcidin upon hepatocyte stimulation with IL‐1β.^31^ A common pathway in regulating the responses to IL‐1β and TLR ligands is not unexpected, as TLRs and IL1R belong to the same superfamily (Toll/IL‐1 receptor [TIR]), which shares a high degree of homology and recruitment of identical adaptor molecules to activate intracellular pathways.^32^, ^33^ Likewise, ERK activation was shown to modulate hepatocyte *Hamp* levels in response to holotransferrin,^34^ but, so far, ERK activation has not been linked to increasing hepatocyte *Hamp* levels in an inflammatory setting. ERK activation appears to be a critical factor in *Hamp* activation in hepatocytes in response to PAMPs, as evidenced by the absence of *Hamp* upregulation in the presence of ERK inhibitors (Figures 3F,H,J and S4H,I) and the observation that other TLR2 ligands not upregulating *Hamp* (Pam3CSK4 and PGN SA) do not induce ERK phosphorylation (Figure S4M). It is interesting to speculate that inflammatory and iron‐controlled responses of hepcidin interact via ERK activation. In macrophages, ERK activation induces *Hamp* expression downstream of TLR4 activation.^35^ The recognition of the ERK pathway as a common regulator of *Hamp* induction upon TLR activation across different cell types highlights a potentially new molecular player in the establishment of hypoferremia, particularly in infectious diseases with increased hepcidin levels in a context of high levels of circulating PAMPs and/or red blood cell breakdown.^3^

We further demonstrate the potential systemic relevance of TLR expression in hepatocytes for the *Hamp* response to PAMPs or pathogens reaching the liver via circulation. Due to its critical position receiving blood from the intestine via the portal vein, the liver is an important firewall for commensal pathogens potentially invading the systemic vasculature. In subjects with liver disease, the firewall function is lost disrupting host‐microbial mutualism due to inefficient clearance.^36^ As expected from innate immune cells, Kupffer cells are highly important for surveillance and as a filtering system.^37^, ^38^ The expression of TLRs by hepatic non‐immune cells, such as hepatocytes, complements Kupffer cell detection and clearance of PAMPs from the blood supply.^39^ The data presented here suggest an important role for hepatocytes in initiating key innate immune responses, such as the immediate upregulation of *Hamp*, once infectious stimuli are filtered through the sinusoidal barrier (Figure 4A,C).

A limitation of this study is the lack of *in vivo* validation of the role of hepatocyte TLR2:TLR6 and TLR5 in mediating the hepcidin response and subsequent hypoferremia during infection. Although previous work demonstrated that FSL1 administration induces hepatic *Hamp* upregulation in WT mice,^40^ definitive evidence would require the generation of mice with hepatocyte‐specific TLR2 deletion in future experiments. Taken together, our results substantiate the critical role of the liver in systemic immune responses by demonstrating the importance of hepatocyte TLRs for the immediate upregulation of *Hamp* in response to PAMPs and pathogens.

## AUTHOR CONTRIBUTIONS

Silvia Colucci, Martina U. Muckenthaler, and Oriana Marques conceived and designed the study. Katharina Bonitz, Silvia Colucci, Ruiyue Qiu, Richard Sparla, Katja Mudder, and Oriana Marques performed experiments. Sandro Altamura and Stefan Zimmermann developed materials/techniques. Katharina Bonitz, Silvia Colucci, Ruiyue Qiu, Sandro Altamura, Matthias W. Hentze, Martina U. Muckenthaler, and Oriana Marques collected and/or analyzed data. Matthias W. Hentze provided expert knowledge and contributed to the critical review of the study. Silvia Colucci, Martina U. Muckenthaler, and Oriana Marques wrote the manuscript. Martina U. Muckenthaler and Oriana Marques acquired funding and supervised the project. All authors revised and accepted the manuscript.

## CONFLICT OF INTEREST STATEMENT

The authors declare no conflict of interest.

### FUNDING

K. B. received support from an Erasmus+ internship. M. U. M. received funding from the DFG (Priority Program SPP2306, GRK2727, and SFB1038), the Federal Ministry of Education and Research (BMBF) (NephrESA Project No. 031L0191C), the Dietmar Hopp‐Stiftung, and the Deutscher Akademischer Austauschdienst (A New Passage to India). M. U. M. and S. A. also received funding from Deutsche Forscungsgemeinschaft: FerrOS ‐ FOR5146; GRK2727; Priority Program SPP2306; SFB1038. O. M. received support from the European Hematology Association (RG66) and an Olympia Morata‐Program Fellowship provided by the Medical Faculty of the University of Heidelberg.

## Supporting information

Supporting information.

## Data Availability

The data generated in this study are available from the corresponding authors on reasonable request.
