# Supplementary material for: Hepatocyte Toll‐like receptors contribute to the hepcidin inflammatory response to pathogens and pathogen‐derived ligands
Source: Hemasphere. 2025 Apr 3;9(4):e70096. doi: 10.1002/hem3.70096 (PMC11966559; doi:10.1002/hem3.70096)
Supplement: Supplementary file 1 — Supporting information. [file HEM3-9-e70096-s001.pdf]

Hepatocyte Toll-like Receptors Contribute to the Hepcidin Inflammatory Response to Pathogens and Pathogen-Derived Ligands

Table S1. Toll-like receptor (TLR) targeted and respective ligands used to activate them.

Ligand	Receptor
Pam3CSK4	TLR2:TLR1
PGN	TLR2
Poly I:C	TLR3
LPS	TLR4
FLA-ST	TLR5
FSL1	TLR2:TLR6
R848	TLR7:TLR8
ODN	TLR9

Table S2. Cytokines detected in the supernatant of FSL1-treated primary hepatocytes. ND, not detected.

Cytokine	Concentration (pg/mL)	
	NT	FSL1
CXCL1	24.80 ± 3.73	432.8 ± 33.2
IL-6	ND	2.14 ± 0.04
TNFα	ND	1.49 ± 0.01

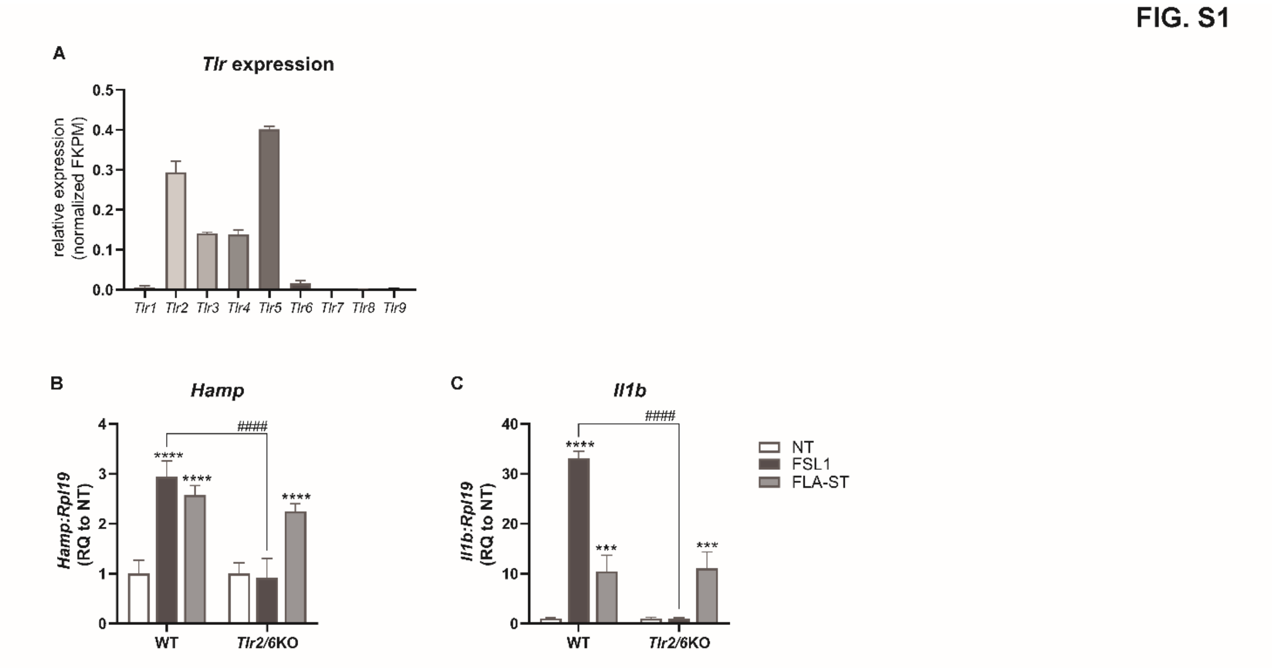

Figure S1. *Tlr2/6*KO hepatocytes retain the capacity to mount a proper inflammatory response to stimulation with the TLR5 ligand FLA-ST. (A) RNA-seq data of primary murine hepatocytes, showing the relative *Tlr1-9* expression. (B,C) Primary murine hepatocytes isolated from WT and *Tlr2/6*KO mice were stimulated for 4 hours with FSL1 or FLA-ST. The mRNA expression of (B) *Hamp* and (C) *Il1b* was analysed by qRT-PCR analysis. mRNA expression data were normalized to the housekeeping gene *Rpl19*. One representative experiment with 4 technical replicates. Data are reported as mean ± SD. Two-way ANOVA, followed by Sidak's multiple comparisons test; */# refers to comparisons with the respective non-treated controls and with the matched treatment in WT controls, respectively: */# p< 0.05, **/## p < 0.01, ***/### p < 0.001, ****/#### p < 0.0001. FKPM, Fragments Per Kilobase of transcript per Million mapped reads; RQ, relative quantification; NT, non-treated; WT, wild-type; Tlr, Toll-like receptor.

FIG.S2

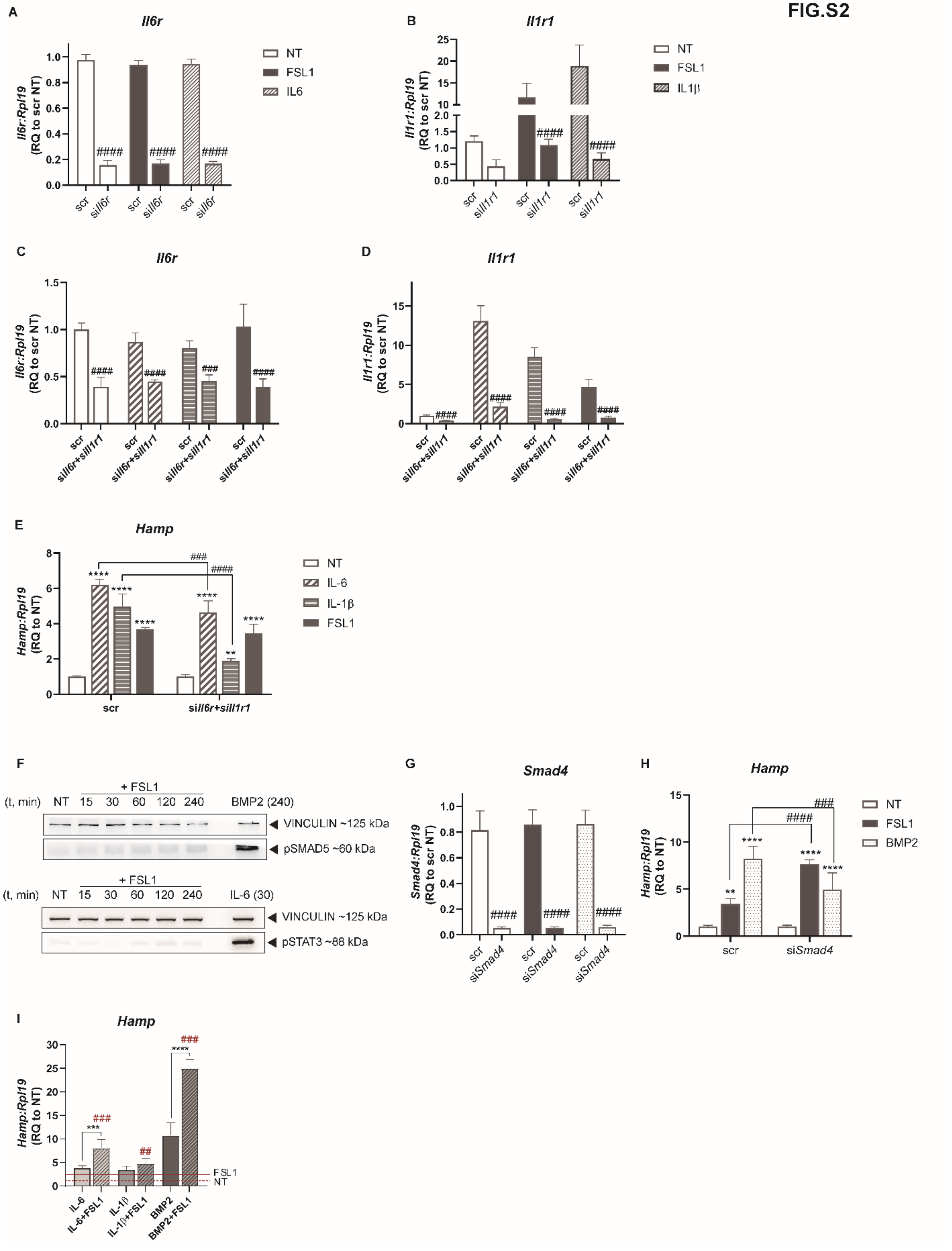

Figure S2. FSL1-mediated *Hamp* upregulation in hepatocytes does not involve the JAK/STAT and BMP/SMAD pathways. (A,B) RNAi for (A) *Il6r* and (B) *Il1r1* was performed in primary hepatocytes for 40

hours, followed by stimulation with FSL1, IL-6 or IL-1β for 4 hours. The mRNA expression of (A) *Il6r* and (B) *Il1r1* was analysed by qRT-PCR analysis. (C-E) RNAi for *Il6r+Il1r1* was performed in primary hepatocytes for 40 hours, followed by stimulation with FSL1, IL-6 or IL-1β for 4 hours. The mRNA expression of (C) *Il6r*, (D) *Il1r1* and (E) *Hamp* was analysed by qRT-PCR analysis. (F) Primary murine hepatocytes were treated with FSL1, BMP2 or IL-6 for the indicated time-points. Western-blot analyses of phospho-SMAD5 and phospho-STAT3. Vinculin was used as loading control. (G,H) RNAi for *Smad4* was performed in primary hepatocytes for 40 hours, followed by stimulation with FSL1 or BMP2 for 4 hours. The mRNA expression of (G) *Smad4* and (H) *Hamp* was analysed by qRT-PCR analysis. (I) Primary hepatocytes were treated with FSL1, IL-6, IL-6 + FSL1, IL-1β, IL-1β + FSL1, BMP2 or BMP2 + FSL1 for 4 hours. The mRNA expression of *Hamp* was analyzed by qRT-PCR. * refers to comparisons with the paired treatment and # to comparisons with FSL1 single treatment. mRNA expression data were normalized to the housekeeping gene *Rpl19*. One representative experiment with 4 technical replicates. Data are reported as mean ± SD. Two-way ANOVA, followed by Sidak's and Dunnett's multiple comparison tests; */# refers to comparisons with the respective non-treated controls and with the matched treatment in scr controls, respectively */# $p < 0.05$, ***/### $p < 0.001$, ****/#### $p < 0.0001$. RQ, relative quantification; NT, non-treated; scr, scrambled.

FIG.S3

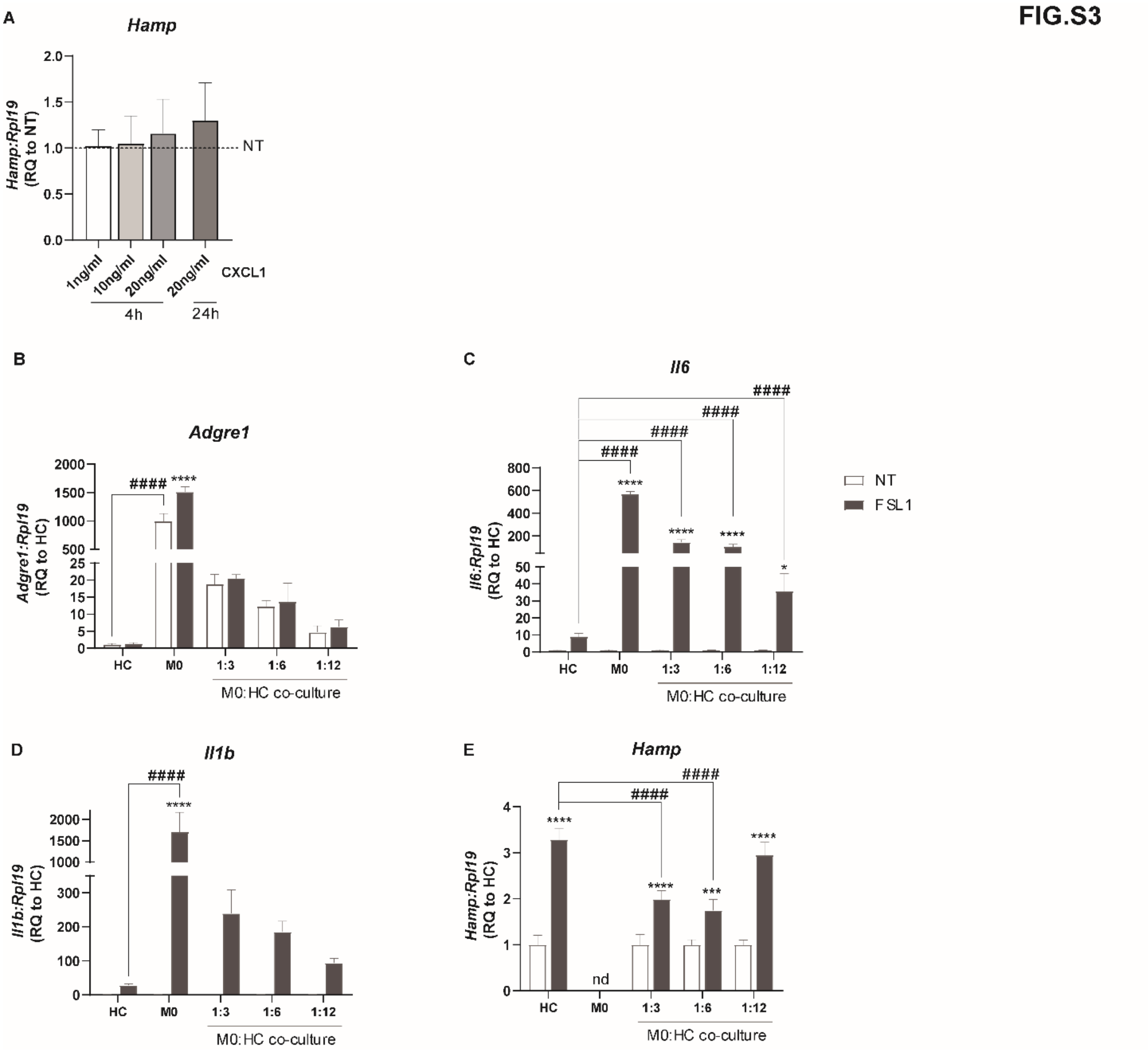

Figure S3. Establishment and characterization of a co-culture of BMDMs and hepatocytes stimulated with FSL1. (A) Primary murine hepatocytes were treated with CXCL1 and collected at the indicated time-points. The mRNA expression of *Hamp* was analysed by qRT-PCR analysis. (B-E) Primary cultures of hepatocytes and BMDMs and co-cultures of BMDMs and hepatocytes at ratios 1:3, 1:6 and 1:12 were stimulated with FSL1 for 4 hours. The mRNA expression of (B) *Adgre*, (C) *Il6*, (D) *Il1b* and (E) *Hamp* were analysed by qRT-PCR. mRNA expression data were normalized to the housekeeping gene *Rpl19*. One representative experiment with 4 technical replicates. Data are reported as mean ± SD. One-way/Two-way ANOVA, followed by Sidak's and Dunnett's multiple comparison tests; */# refers to comparisons with the respective non-treated controls and with the matched treatment in HC controls, respectively */# $p < 0.05$, ***/### $p < 0.001$, ****/#### $p < 0.0001$. RQ, relative quantification; NT, non-treated; M0, macrophage; HC, hepatocyte; nd, non-detectable.

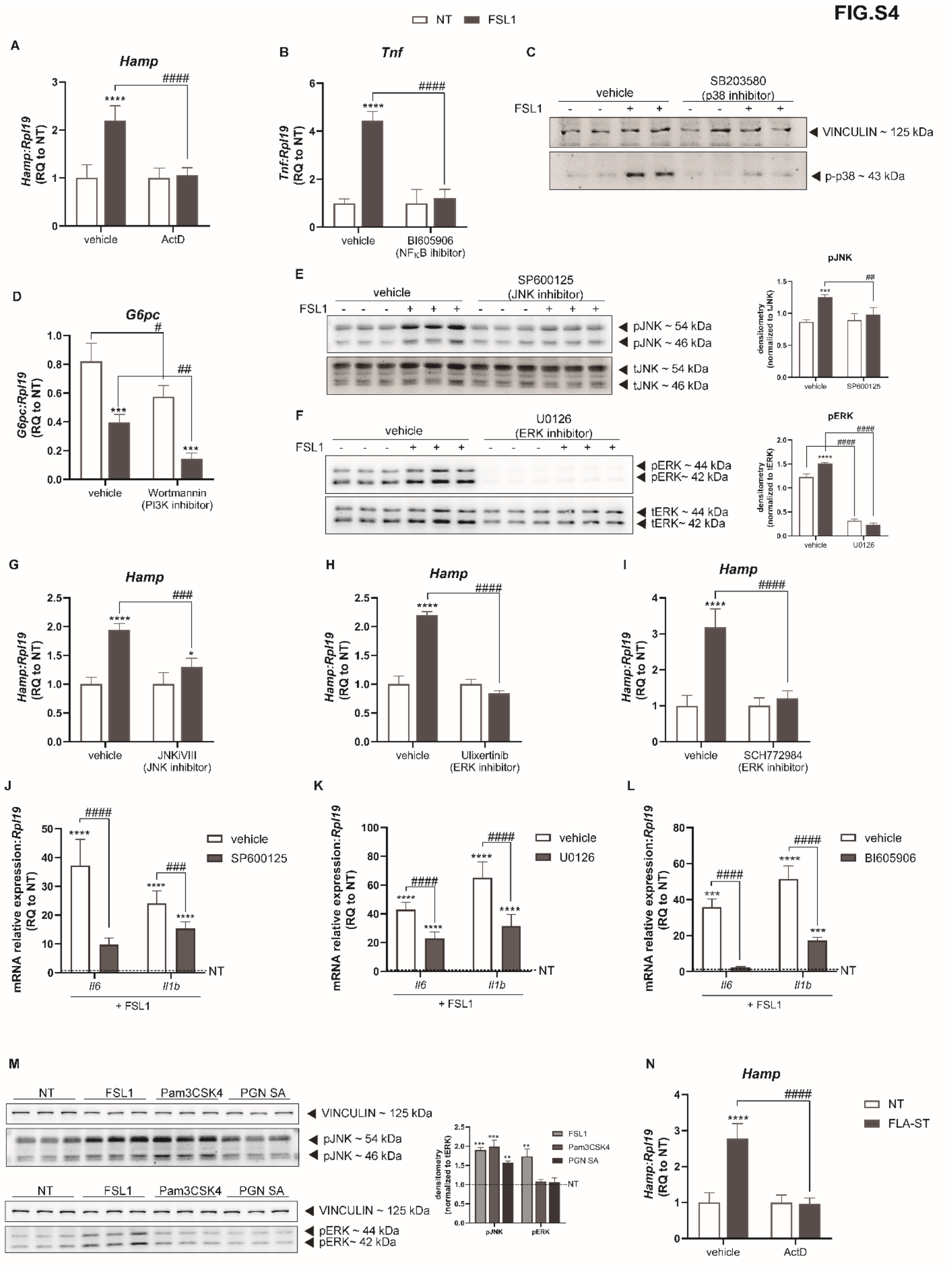

Figure S4. Inhibition of signaling pathways downstream TLR activation under stimulation with FSL1. (A) Primary murine hepatocytes were pre-treated for one hour with Actinomycin D (ActD) and then

stimulated with FSL1 for 4 hours. The mRNA expression of *Hamp* was analysed by qRT-PCR. (B) Primary murine hepatocytes were pre-treated for one hour with BI605906 and then stimulated with FSL1 for 4 hours. The mRNA expression of *Tnf* was analysed by qRT-PCR. (C) Primary murine hepatocytes were pre-treated for one hour with SB203580 and then stimulated with FSL1 for 30 minutes. Western-blot analyses of phospho-p38. Vinculin was used as loading control. (D) Primary murine hepatocytes were pre-treated for one hour with Wortmannin and then stimulated with FSL1 for 4 hours. The mRNA expression of *G6pc* was analysed by qRT-PCR analysis. (E,F) Primary murine hepatocytes were pre-treated for one hour with (E) SP600125 or (F) U0126 and then stimulated with FSL1 for 30 minutes. Western-blot analysis of (E) phospho-JNK and (F) phospho-ERK, with total JNK and ERK as loading controls, respectively. (G-I) Primary murine hepatocytes were pre-treated for one hour with (G) JNKiVIII, (H) Ulixertinib or (I) SCH772984 and then stimulated with FSL1 for 4 hours. The mRNA expression of *Hamp* was analysed by qRT-PCR analysis. (J-L) Primary murine hepatocytes were pre-treated for one hour with (J) SP600125, (K) U0126 or (I) BI605906 and then stimulated with FSL1 for 4 hours. The mRNA expression of *Il6* and *Il1b* was analysed by qRT-PCR analysis. (M) Primary murine hepatocytes were pre-treated for one hour with SP600125 or U0126 and then stimulated with FSL1, Pam3CSK4 or PGN SA for 30 minutes. Western-blot analyses of phospho-JNK and phospho-ERK. Vinculin was used as loading control. (N) Primary murine hepatocytes were pre-treated for one hour with Actinomycin D (ActD) and then stimulated with FLA-ST for 4 hours. The mRNA expression of *Hamp* was analysed by qRT-PCR. mRNA expression data were normalized to the housekeeping gene *Rpl19*. One representative experiment with 4 technical replicates. Data are reported as mean ± SD. Two-way ANOVA, followed by Sidak's and Dunnett's multiple comparison tests; */# refers to comparisons with the respective non-treated controls and with the matched treatment in vehicle controls, respectively: */# $p < 0.05$, **/## $p < 0.01$, ***/### $p < 0.001$, ****/#### $p < 0.0001$. RQ, relative quantification; NT, non-treated; p, phospho; t, total.

FIG.S5

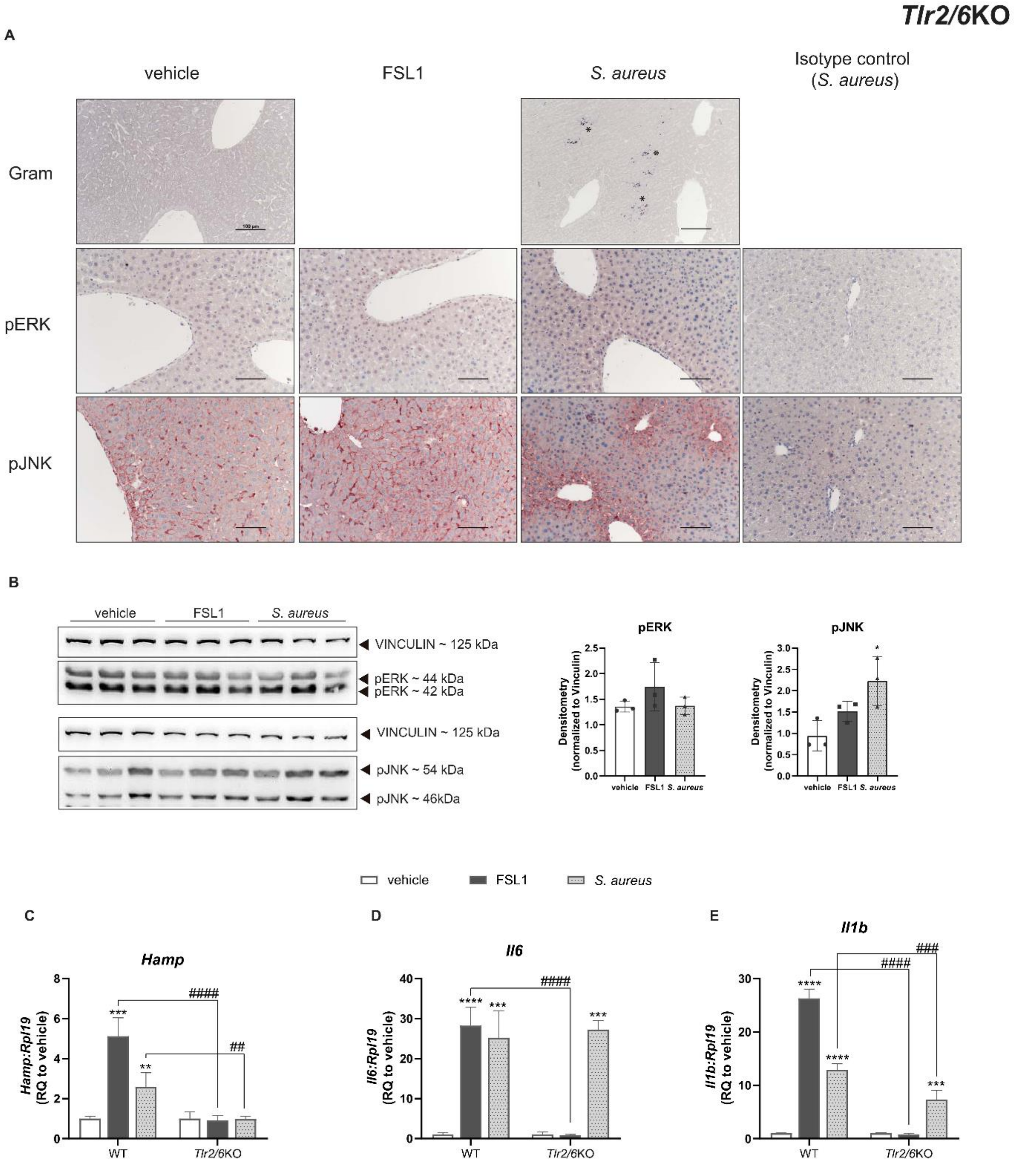
Tlr2/6KO
A
vehicle
FSL1
S. aureus
Isotype control (S. aureus)
Gram
pERK
pJNK
B
vehicle
FSL1
S. aureus
VINCULIN ~ 125 kDa
pERK ~ 44 kDa
pERK ~ 42 kDa
VINCULIN ~ 125 kDa
pJNK ~ 54 kDa
pJNK ~ 46kDa
pERK
pJNK
Densitometry (normalized to Vinculin)
vehicle
FSL1
S. aureus
vehicle
FSL1
S. aureus
C
D
E
Hamp
Hamp:Rpl19 (RQ to vehicle)
Il6
Il6:Rpl19 (RQ to vehicle)
Il1b
Il1b:Rpl19 (RQ to vehicle)
WT
Tlr2/6KO

Figure S5. *Ex vivo* perfusion of *Tlr2/6*KO mice livers with FSL1 or heat-killed *S. aureus*. (A) Livers from *Tlr2/6*KO mice were perfused with FSL1 or heat-killed *S. aureus* and then further processed. Liver slices were fixed in 4%PFA, embedded in paraffin and cut at 5 μm thickness. Sections were stained for Gram or processed for immunohistochemical analysis of phospho-ERK and phospho-JNK. Asterisks indicate bacterial aggregates (B) After perfusion, livers pieces were collected. Western-blot analyses of phospho-JNK and phospho-ERK. Vinculin was used as a loading control and for data normalization. One-way ANOVA followed by Dunnett's multiple comparison test. * refers to comparisons with the non-treated control. (C-E) Hepatocytes derived from the livers of WT and *Tlr2/6*KO mice perfused with FSL1 or heat-killed *S. aureus* were plated and collected after 4 hours. The mRNA expression of (C) *Hamp*, (D) *Il6* and (E) *Il1b* were analysed by qRT-PCR analysis. mRNA expression data were normalized to the housekeeping gene *Rpl19*. One representative experiment with 4 technical replicates. Data are reported as mean ± SD. Two-way ANOVA, followed by Sidak's and Dunnett's multiple comparison tests; */# refers to comparisons with the respective vehicle-treated controls and with the matched treatment in WT controls, respectively **/## $p < 0.01$, ***/### $p < 0.001$, ****/#### $p < 0.0001$. p, phospho; t, total; RQ, relative quantification.
